# ECG‐based cardiodynamicsgram can reflect anomalous functional information in coronary artery disease

**DOI:** 10.1002/clc.24019

**Published:** 2023-04-06

**Authors:** Ying Wang, Jiaxin Sun, Kui Sun, Lin Li, XinXin Yu, Cong Wang, Hui Gu, Qinghua Sun, Ximing Wang

**Affiliations:** ^1^ Department of Radiology, Shandong Provincial Hospital Shandong University Jinan China; ^2^ Center for Intelligent Medical Engineering, School of Control Science and Engineering Shandong University Jinan China; ^3^ Department of Radiology Shandong Provincial Hospital Affiliated to Shandong First Medical University Jinan China; ^4^ Department of Cardiology Shandong Provincial Hospital Affiliated to Shandong First Medical University Jinan China

**Keywords:** cardiodynamicsgram, computed tomography‐derived fractional flow reserve, coronary artery disease, coronary computed tomography angiography, electrocardiogram

## Abstract

**Background:**

The cardiodynamicsgram (CDG), a novel noninvasive method, extracts dynamic ST‐T segment information from an electrocardiogram (ECG) through deterministic learning.

**Hypothesis:**

The CDG can reflect anomalous functional information in coronary artery disease (CAD).

**Methods:**

We retrospectively enrolled 456 patients with suspected CAD who underwent coronary computed tomography angiography (CCTA) from January 2020 to 2022, followed immediately by standard 12‐lead ECG acquisition. Positivity for CAD were defined as CCTA ≥ 50% or CT‐derived fractional flow reserve (CT‐FFR) ≤ 0.8. A CDG value <0 was considered negative; otherwise, it was considered positive. We also evaluated the diagnostic performance of the CDG in the ECG‐diagnosis‐negative subgroup and in patients who had undergone invasive coronary angiography (ICA) after CCTA.

**Results:**

Of 362 patients, 168 (46.41%) were positive for CAD, and 178 (49.17%) were men. The median age was 59 (52−66) years. The accuracy of the CDG in the diagnosis of CAD was 79.56%, with a sensitivity, specificity, and the area under the receiver operating characteristic curve (AUC) of 75.60%, 82.99%, and 0.836 (95% CI: 0.794−0.878), respectively. Similarly, in the ECG‐diagnosis–negative subgroup (*n* = 223), the accuracy of the CDG was 80.27%, with an AUC of 0.842 (95% CI: 0.790−0.895). Among the 11 patients with CAD confirmed by ICA, 10 were diagnosed positive by the CDG. Furthermore, the CDG values and CT‐FFR were correlated (*r* = −.395; *p* < .001).

**Conclusions:**

The ECG‐based CDG has relatively high specificity and accuracy for the diagnosis of CAD and reflects functional cardiac information to some extent. It has the potential to be used as a screening tool for suspected CAD patients before CCTA.

## Graphical abstract

In this study, using coronary computed tomography angiography and CT‐fractional flow reserve as reference standards, the electrocardiogram‐based cardiodynamicsgram was found to have relatively high specificity and accuracy for the diagnosis of coronary artery disease.

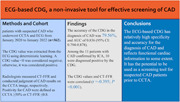

**ABBREVIATIONS**
CAD: coronary artery disease
CCTA: coronary computed tomography angiography
CDG: cardiodynamicsgram
CT‐FFR: computed tomography‐derived fractional flow reserve
ECG: electrocardiogram
ICA: invasive coronary angiography

## 1 INTRODUCTION

Coronary artery disease (CAD) is one of the most common cardiovascular diseases leading to death in the global population.^1^ Coronary atherosclerosis is the primary reason for CAD, which manifests as progressive occlusion of the coronary arteries with consistently lower oxygen and nutrient perfusion.^2^ Asymptomatic, stable plaques can also become unstable at any time due to plaque rupture or erosion and even directly lead to fatal events.^3^

Coronary computed tomography angiography (CCTA) is the preferred diagnostic test for CAD,^4^ used to assesses the position and extent of atherosclerotic plaque and the degree of coronary artery stenosis. CCTA has the imaging advantages of more availability and less invasiveness. However, it is radioactive, and the severe allergic reactions and kidney damage caused by contrast injection cannot be ignored.^3^, ^5^ Moreover, CCTA provides only anatomical information on the coronary arteries, and coronary stenosis are often overestimated, only a minority of severe stenoses diagnosed by CCTA are finally recognized as myocardial ischemia.^6^ To obtain functional information requires complex postprocessing operations on CCTA images, such as CT‐derived fractional flow reserve (CT‐FFR). The CT‐FFR is a noninvasively obtained parameter used to assess hemodynamically significant stenoses without the need for additional testing and radiation exposure, and CT‐FFR accuracy of 90% compared to invasive FFR.^7^, ^8^, ^9^

Clinically, the electrocardiogram (ECG) is the most common test for patients with suspected CAD.^10^ However, interpretation of the ECG signal is highly influenced by the observers, and fluctuations in the ECG signals generated by early CAD are too subtle to detect.^11^ Deterministic learning has been proposed for the accurate modeling and rapid recognition of temporal or dynamical patterns.^12^, ^13^, ^14^ The cardiodynamicsgram (CDG) is a new noninvasive tool for detecting subtle changes in the ST‐T segment of the ECG. The CDG can extract quantitative dynamic information on its spatial and temporal dispersion by deterministic learning and be visualized with a three‐dimensional (3D) image to illustrate the abnormalities of the heart. Recently, some studies have confirmed that the CDG can predict ≥50% of coronary stenosis and has exceptional diagnostic performance for myocardial ischemia.^15^ However, no study has evaluated the ability of CDG to reflect abnormal cardiac function and thus aid in the screening of CAD.

Precise, easy, rapid, and noninvasive tools for screening CAD in the clinic are urgently needed. Hence, this study attempted to evaluate the diagnostic performance of the CDG in detecting CAD using CCTA and CT‐FFR as reference standards. We also analyzed the correlation between CDG values and CT‐FFR.

## 2 MATERIALS AND METHODS

### 2.1 Study design and population

**Figure 1:**
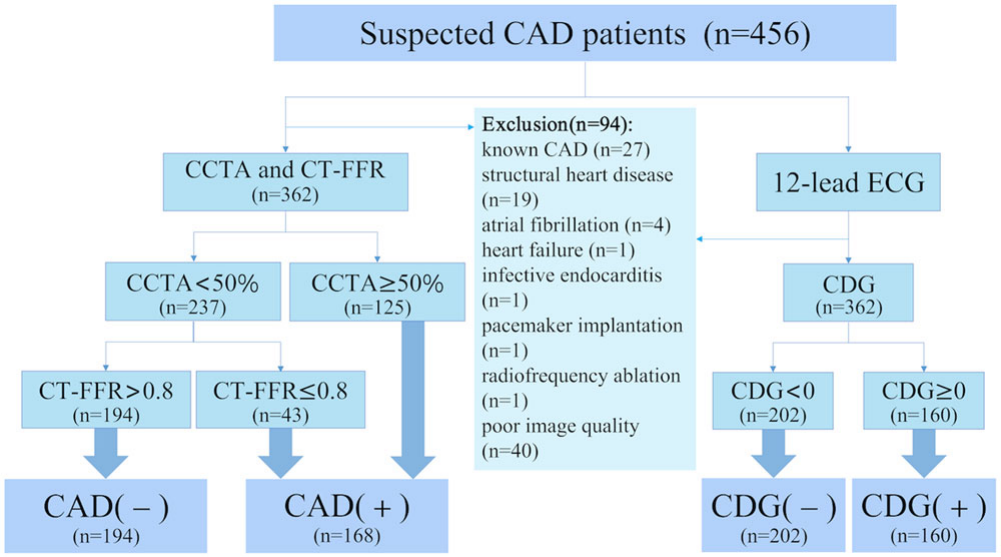
Flow chart of the patient population included in this study. CAD (−): stenosis <50% on CCTA and CT‐FFR > 0.8; CAD (+): stenosis ≥50% on CCTA or CT‐FFR ≤ 0.8; CDG (−): CDG < 0; CDG (+): CDG ≥ 0. CAD, coronary artery disease; CCTA, coronary computed tomography angiography; CDG, cardiodynamicsgram; CT‐FFR, computed tomography‐derived fractional flow reserve.

This retrospective study included 456 patients with suspected CAD who underwent CCTA at Shandong Provincial Hospital from January 2020 to 2022. All patients underwent standard 12‐lead ECG examination immediately after a CCTA scan. Patients were excluded if they had known CAD (including previous myocardial infarction, coronary artery bypass grafting, and percutaneous coronary stent implantation), structural heart disease (including myocardial diseases, valvular pathology more than mild, congenital heart disease), atrial fibrillation, heart failure, infective endocarditis, pacemaker implantation, radiofrequency ablation, or poor image quality (Figure 1). The reference standard for CAD positivity was stenosis on CCTA ≥ 50% or CT‐FFR ≤ 0.8.^16^, ^17^ All parameters in this study were performed blinded to each other and clinical outcomes, and did not influence clinical treatment. The study was approved by the Ethics Committee of Shandong Provincial Hospital, and the requirement for informed consent was waived.

### 2.2 CCTA image acquisition

Third‐generation dual‐source CT (Siemens SOMATOM Force; Siemens Healthcare) was used for image acquisition. Before scanning, patients (except for patients with hypotension) were treated with a sublingually applied nitroglycerine pump spray to dilate the coronary arteries and received breath‐hold training to reduce respiratory motion artifacts.

The nonionic contrast agents iohexol 30.0−55.0 mL and 0.9% sodium chloride injection 30.0−55.0 mL were injected into the peripheral vein on the back of the hand at a flow rate of 4.0 mL/s using a double‐barrel autoinjector. The aortic root was selected as the region of interest (ROI), and the CT attenuation value was monitored in the ROI. The CCTA scan was automatically triggered with a 5 s delay when the CT attenuation value reached 100 HU. The scanning area was from the aortic arch 1−1 cm below the diaphragmatic surface of the heart.

The scan parameters were as follows: collimator 192 × 0.6 mm, layer thickness 0.75 mm, rotation time 0.25 s/turn, and tube voltage 70−120 kV. A prospective or retrospective ECG gated spiral scan mode was selected depending on the patient's heart rate and respiratory control.

### 2.3 CCTA image analysis

For analysis, all images were transferred to a Siemens workstation with semiautomatic image segmentation software (Syngo.via; VB20A; Siemens Healthcare). The axial image reconstruction section thickness was 0.75 mm, the reconstruction increment was 0.5 mm, and the smoothed convolution kernel was Bv40. Image postprocessing techniques include maximum intensity projection, multiplane reconstruction, curved plannar reconstruction, and volume rendering. The results of CCTA are diagnosed by two specialists with 10 years of experience in diagnostic cardiovascular imaging, respectively. When there is a disagreement, the two specialists discuss the decision. Stenosis severity was classified into the following groups: 0%, 1%−24%, 25%−49%, 50%−69%, 70%−99%, and 100%. CCTA results were defined as positive if at least one epicardial coronary artery with a stenotic degree ≥50% and diameter ≥2.0 mm.^18^

### 2.4 CT‐FFR values acquisition

CT‐FFR analysis was performed using cFFR software (version 3.2.5; Siemens Healthcare). This software is based on a deep learning model and predicts the FFR values of coronary arteries. After importing the CCTA images into the software, the coronary centerline and lumen were automatically identified and later manually corrected if necessary. Then, a coronary tree was generated with different colors representing different CT‐FFR values. The CT‐FFR was measured for all vessels of diameter ≥1.8 mm in the coronary tree, the lesion‐specific CT‐FFR (defined as the per‐patient lowest CT‐FFR value 2 cm distal to lesion) was recorded in patients with vascular stenosis, and the vessel‐specific CT‐FFR (defined as the per‐patient lowest CT‐FFR value in the distal part of vessels) was recorded in patients without plaques.^16^ Occluded vessels were assigned a value of 0.5.^19^ CT‐FFR results were defined as positive if the CT‐FFR value ≤0.8.^18^

### 2.5 Invasive coronary angiography (ICA)

Transracial artery puncture was chosen for the ICA procedure. Iodixano was used as a contrast agent. Stenosis severity was classified as follows: 0%, 1%−24%, 25%−49%, 50%−69%, 70%−99%, and 100%. ICA results were defined as positive if at least one epicardial coronary artery had stenosis ≥50% and diameter ≥2.0 mm.

### 2.6 Standard 12‐lead ECG

The digital 12‐lead ECG information of the patients was recorded using an electrocardiograph (Mindray Bene‐Heart R12) with a 1000 Hz sampling rate and 0.1 µV resolution. Participants were in the resting state and supine position. The ECG data were recorded for at least 20 s for further analysis. The 12‐lead ECG was diagnosed with ischemic changes if any of the following conditions were present, and the patient was considered positive: (1) New ST‐elevation: ≥0.2 mV (men) or ≥0.15 (women) in chest leads V2−V3, and/or ≥0.1 mV in all leads other than V2−V3; (2) New horizontal or down‐sloping ST‐depression ≥0.05 mV in two adjacent leads; (3) T‐wave inversion ≥0.1 mV in two adjacent leads with obvious R‐wave or R/S > 1. The ECG results are diagnosed by two specialists with 10 years of experience in ECG diagnosis, respectively. When there is a disagreement, the two specialists discuss the decision.

### 2.7 CDG and feature extraction

The CDG was generated as follows. First, the digital 12‐lead ECG were filtered to eliminate interference such as power frequency(50 Hz), baseline drift, motion artifact, EMG, and so on. Then, we transformed the 12‐lead ECG signals into the 3‐lead ECG vector signals by linear transformation matrix of Kors.^20^ Based on the lead signal of R wave as the main wave in the vector ECG signals, the R wave peak, J point, T wave apex and T wave endpoint were located, and the ST‐T loops in the vector ECG signals were obtained by intercepting the ST‐T segments representing the ventricular repolarization process.^21^ After that, the ST‐T loop of the vectorcardiogram was modeled using a Radial Basis Function neural network to obtain the dynamic ECG information. The CDG was generated by plotting the extracted cardiodynamics information as a 3D graph. The shapes of the CDG represent the dispersion of cardiac repolarization, which is closely related to the degree of myocardial ischemia.^15^ These shapes have been shown to be remarkably different between ischemia patients and healthy controls, with the latter presenting with a noticeably regular or annular shape, while CAD patients demonstrate an irregular or nonannular shape.

Then, the shapes of the CDG were interpreted by evaluating the spatial characteristics based on the Lyapunov index and the temporal characteristics based on the Fourier transform of the CDG.^22^ The spatial heterogeneity index (SHI) and temporal heterogeneity index (THI) were defined as follows:

$$\text{SHI}\;=\frac{1}{N}\sum_{n=1}^{N}\ln(d_{n2}/d_{n1})$$

$$\text{THI}\;={\text{argmin}}_{\lambda_{i}}|F\cdot \text{exp}(-0.1\lambda_{i})|$$

where *N* represents the number of data points in the CDG, *d*_*n1*_ represents the distance between the nth data point and its nearest data point, *d*_*n2*_ represents the distance between the nth data point and its nearest data point after 10 steps, and *F* represents the Fourier transform of the CDG. Finally, a linear support vector machine was used as a classifier to train the screening model for CAD. The CDG value is defined as the distance between the SHI and THI and the classification boundary^23^ and was calculated as follows:

$${\mathrm{CDG}}_{\mathrm{value}}=-0.0556\mathrm{\cdot THI}\;+30.8131\mathrm{\cdot SHI}-2.7719$$

CDG value is calculated automatically using the computer program by data analyst who is blind to clinical information.

According to the data of patients with normal or roughly normal chest pain from previous studies, a linear classification boundary was obtained from a CAD detection model established by using machine learning algorithm of linear kernel SVM as follows:

$${\mathrm{CDG}}_{\mathrm{boundary}}=-0.0556\mathrm{\cdot THI}\;+30.8131\mathrm{\cdot SHI}-2.7719=0$$

The distance of the case samples to the linear classification boundary was a CDG value, the judgment standards were a CDG value ≥0 for positive and <0 for negative.

### 2.8 Statistical analysis

The Kolmogorov−Smirnov test was used to check the assumption of a normal distribution. Normally distributed variables are expressed as the means ± standard deviations. Nonnormally distributed variables are expressed as the median (IQR). Student's *t*‐test was used for normally distributed data, and the Mann−Whitney *U* test was used for nonnormally distributed data. Categorical variables are presented as counts (percentages) and were compared using the *χ* ^2^ test or Fisher's exact test. The performance of the CDG and standard 12‐lead ECG in diagnosing CAD were assessed using the area under the receiver operating characteristic curve (AUC), accuracy, sensitivity, and specificity. The relationship between CDG values and CT‐FFR was assessed by using Spearman's correlation analysis. The reproducibility of CT‐FFR values was tested using interclass correlation coefficient. A two‐tailed *p* value <.05 was considered statistically significant. All statistical analyses were conducted using statistical software (IBM SPSS Statistics; version 26.0).

## 3 RESULTS

### 3.1 Baseline characteristics

A total of 362 patients with suspected CAD were included, with a median age of 59 (52−66) years, and 178 (49.17%) were male. A total of 94 (20.61% [94/456]) patients were excluded. A baseline comparison showed no significant differences in sex, body mass index, hypertension, diabetes, hyperlipidemia, or smoking history between positive and negative CDG patients. However, positive patients were older (*p* < .001) and more likely to have hypertension (*p* = .002) than negative patients (Table 1). The degree of coronary stenosis was ≥50% in 125 (34.53%) patients, and the CT‐FFR values were ≤0.8 in 153 (42.26%) patients (Table 2).

**Table 1:** Patient demographics.

Characteristic	All (*n* = 362)	CAD (−) (*n* = 194)	CAD (+) (*n* = 168)	*p*
Age, year	59.0 (52.0−66.0)	57.0 (47.0−65.0)	62.5 (56.0−68.0)	<.001
Male (%)	178/362 (49.17)	87/194 (44.85)	91/168 (54.17)	.077
Risk factors				
Hypertension (%)	82/147 (55.78)	28/67 (41.79)	54/80 (67.50)	.002
Diabetes (%)	32/147 (21.77)	10/67 (14.93)	22/80 (27.50)	.067
Hyperlipidemia (%)	62/147 (42.18)	33/67 (49.25)	29/80 (36.25)	.113
Smoking history (%)	41/147 (27.89)	15/67 (22.39)	26/80 (32.50)	.175
BMI, kg/m^2^	25.61 (23.42−27.99)	25.41 (23.86−27.95)	25.71 (23.24−28.23)	.751
Stenosis extent on CCTA, %				<.001
0 (%)	95/362 (26.24)	86/194 (44.33)	9/168 (5.36)	
1−24 (%)	66/362 (18.23)	59/194 (30.41)	5/168 (2.98)	
25−49 (%)	76/362 (20.99)	49/194 (25.26)	29/168 (17.26)	
50−69 (%)	65/362 (17.96)	0	65/168 (38.69)	
≥70 (%)	48/362 (13.26)	0	48/168 (28.57)	
100 (%)	12/362 (3.31)	0	12/168 (7.14)	
CT‐FFR	0.82 (0.65−0.89)	0.88 (0.85−0.91)	0.63 (0.43−0.74)	<.001
>0.8 (%)	209/362 (57.73)	194/194 (100)	15/168 (8.93)	
≤0.8 (%)	153/362 (42.27)	0	153/168 (91.07)	
CDG	−0.55 (−2.77 to 1.15)	−2.24 (−3.84 to −0.59)	0.87 (0.03−2.21)	<.001
CDG < 0 (%)	202/362 (55.80)	161/194 (82.99)	41/168 (24.40)	
CDG ≥ 0 (%)	160/362 (44.20)	33/194 (17.01)	127/168 (75.60)	

*Note*: Data are presented as the median (IQR) or frequency and percentages as appropriate. CAD (−): stenosis <50% on CCTA and CT‐FFR >0.8; CAD (+): stenosis ≥50% on CCTA or CT‐FFR ≤0.8.

Abbreviations: BMI, body mass index; CAD, coronary artery disease; CCTA, coronary computed tomography angiograph; CDG, cardiodynamicsgram; CT‐FFR, computed tomography‐derived fractional flow reserve.

**Table 2:** Comparison of the CDG with CCTA and CT‐FFR.

	CCTA < 50%		CCTA ≥ 50%		Total
	CT‐FFR > 0.8	CT‐FFR ≤ 0.8	CT‐FFR > 0.8	CT‐FFR ≤ 0.8	
CDG < 0 (%)	161 (44.47)	7 (1.93)	6 (1.66)	28 (7.73)	202 (55.80)
CDG ≥ 0 (%)	33 (9.12)	36 (9.94)	9 (2.49)	82 (22.65)	160 (44.20)
Total (%)	194 (53.59)	43 (11.88)	15 (4.14)	110 (30.39)	362

*Note*: Data are presented as frequencies and percentages as appropriate.

Abbreviations: CCTA, coronary computed tomography angiograph; CDG, cardiodynamicsgram; CT‐FFR, computed tomography‐derived fractional flow reserve.

### 3.2 Diagnostic performance of the CDG

**Figure 2:**
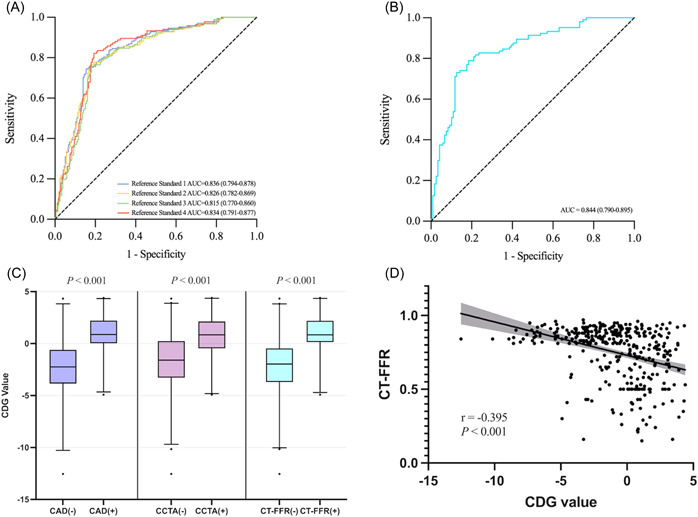
Receiver operating characteristic (ROC) curve analysis for different groups, and association between cardiodynamicsgram (CDG) values and different parameters. (A) The area under the receiver operating characteristic curve (AUC) for the diagnosis of coronary artery disease (CAD) by the CDG for all included patients (*n* = 362) at different thresholds. (B) The AUC for the diagnosis of CAD by the CDG was 0.842 for the subgroup (*n* = 223) with a negative standard 12‐lead electrocardiogram diagnosis. (C) Grouped using different criteria, the positive subgroups all showed higher CDG values than the negative subgroups. (D) Correlation analysis of the CDG value and computed tomography‐derived fractional flow reserve (CT‐FFR). Reference standard 1: Positivity for CAD were defined as CCTA ≥ 50% or CT‐FFR ≤ 0.8; Reference standard 2: Positivity for CAD were defined as CCTA ≥ 50% or CT‐FFR ≤ 0.7; reference standard 3: Positivity for CAD were defined as CCTA ≥ 70% or CT‐FFR ≤ 0.8; reference standard 4: Positivity for CAD were defined as CCTA ≥ 70% or CT‐FFR ≤ 0.7. CAD (−): stenosis <50% on CCTA and CT‐FFR > 0.8; CAD (+): stenosis ≥50% on CCTA or CT‐FFR ≤ 0.8; CCTA (−): stenosis <50% on CCTA; CCTA (+): stenosis ≥50% on CCTA; CT‐FFR (−): CT‐FFR > 0.8; CT‐FFR (+): CT‐FFR ≤ 0.8. CCTA, coronary computed tomography angiography; CT‐FFR, computed tomography‐derived fractional flow reserve.

There were 168 (46.41%) positive and 194 (53.59%) negative CAD patients. Using CCTA and CT‐FFR as a reference standard, the CDG achieved an accuracy of 79.56%, sensitivity of 75.60%, and specificity of 82.99% in diagnosing CAD, and the corresponding AUC was 0.836 (95% CI: 0.794−0.878; Figure 2A). Compared with the physician's diagnostic results with standard 12‐lead ECG, the CDG improved the accuracy by 29.01 percentage points and the sensitivity, specificity, and AUC by 37.50 percentage points, 21.65 percentage points, and 0.330, respectively (Figure 2B). False positive ratings were greatly reduced, observed in 33 (17.01%) patients with the CDG versus 75 (36.76%) patients with the ECG. We also analyzed the diagnostic performance of CDG at lower thresholds (Table 3).

**Table 3:** Diagnostic performance of the standard 12‐lead ECG and CDG.

	*n*	TP	TN	FP	FN	Sensitivity (%)	Specificity (%)	PPV (%)	NPV (%)	Accuracy (%)	AUC
ECG	362	64	119	75	104	38.10	61.34	46.04	53.36	50.55	0.497
CDG	362										
Reference standard 1		127	161	33	41	75.60	82.99	79.38	79.70	79.56	0.836
Reference standard 2		119	168	41	34	77.78	80.38	74.38	83.17	79.28	0.826
Reference standard 3		120	165	40	37	76.43	80.49	75.00	81.68	78.72	0.815
Reference standard 4		112	180	48	22	83.58	78.95	70.00	89.11	80.66	0.834

Abbreviations: AUC, area under the curve; CAD, coronary artery disease; CCTA, coronary computed tomography angiography; CDG, cardiodynamicsgram; CT‐FFR, computed tomography‐derived fractional flow reserve; ECG, electrocardiogram; FN, false negative; FP, false positive; NPV, negative predictive value; PPV, positive predictive value; TN, true negative; TP, true positive.

Reference standard 1: Positivity for CAD were defined as CCTA ≥ 50% or CT‐FFR ≤ 0.8. Reference standard 2: Positivity for CAD were defined as CCTA ≥ 50% or CT‐FFR ≤ 0.7. Reference standard 3: Positivity for CAD were defined as CCTA ≥ 70% or CT‐FFR ≤ 0.8. Reference standard 4: Positivity for CAD were defined as CCTA ≥ 70% or CT‐FFR ≤ 0.7.

A total of 223 (61.60%) had a negative ECG presentation. In these patients, the accuracy of the CDG in diagnosing CAD was 80.27%, with a sensitivity and specificity of 74.04% and 85.71%, respectively, and an AUC of 0.842 (95% CI: 0.790−0.895; Supporting Information: Table S1).

In this study, 13 (3.59%) patients underwent ICA, and the interval between ICA and CCTA ranged from 3 to 59 days. Among them, 2 patients had a degree of stenosis on ICA < 50% (1 CDG positive, 1 CDG negative), and 11 patients had ICA ≥ 50% (10 CDG positive, 1 CDG negative).

### 3.3 CDG value analysis

We found that the positive CAD group had a significantly higher CDG value than the negative CAD group, 0.87 (0.03−2.21) versus −2.24 (−3.84 to −0.59), *p* < .001. When stenosis on CCTA ≥ 50% was used as a reference, the CDG value of 0.84 (−0.46 to 2.13) in the positive CAD group was significantly higher than the −1.60 (−3.28 to 0.24) in the negative CAD group (*p* < .001). When CT‐FFR ≤ 0.8 was used as a reference, the CDG value of 0.84 (0.14−2.20) in the positive CAD group was still higher than the −1.97 (−3.70 to −0.46) in the negative CAD group (*p* < .001; Figure 2C). Additionally, the CDG value and CT‐FFR were correlated (*r* = −.395; *p* < .001; Figure 2D). Supporting Information: Table S2 show the CT‐FFR values had excellent reproducibility, and Supporting Information: Figures S1 and S2 show examples of patients who were negative and positive for CAD.

## 4 DISCUSSION

According to the 2016 update of the stable chest pain guideline, the National Institute for Health and Care Excellence recommends that CCTA be the first‐line test for stable chest pain patients or for nonanginal chest pain patients with ECG changes suggesting underlying CAD.^10^, ^24^, ^25^ Nevertheless, CCTA demonstrates several disadvantages. First, the examination fee is much higher than that of ECG. Second, patients are at risk of radiation exposure, which is forbidden for pregnant women.^26^ Although CT‐FFR can provide hemodynamic information within the vessels, it is time consuming due to the need to manually adjust the centerline and boundary of the lumen and calculate multivessel fractions.^27^ The ECG‐derived CDG can also provide functional information, but the calculation time from deterministic learning is far shorter than that of CT‐FFR. In our study, we discovered that the ECG‐based CDG performed very well in diagnosing suspected CAD populations. This method has potential value in supporting physicians' decisions in the clinical setting.

Diagnosing with the standard 12‐lead ECG mainly depends on the physician's visual assessments, resulting in a certain degree of subjectivity, and tiny changes are frequently missed.^11^, ^28^ It has been shown that subtle changes in the ST‐T segment of the ECG signal can reflect cardiac function and myocardial injury.^29^, ^30^ The CDG, based on deterministic learning theory, focuses on analyzing these subtle abnormal signals and detecting the dynamics of cardiac repolarization.^31^ This cardiac dynamic information represents spatial and temporal variations in the repolarization phase of cardiac activation versus the static characteristics of the ECG signal. The CDG contains deeper information and can capture more sensitive changes than standard ECG. The results show that the diagnostic performance of CDG was much better than that of standard ECG, with an AUC of 0.836 (0.794−0.878) versus 0.497 (0.437−0.557). The CDG was also superior to the ECG in terms of positive and negative detection rates, with positive predictive values (PPVs) of 79.38% (127/160) versus 46.04% (64/139) and negative predictive values of 79.70% (161/202) versus 53.36% (119/223). Therefore, the CDG can be used as a noninvasive diagnostic tool for the early detection of suspected CAD before significant pathological changes appear on the ECG. In the group with a negative ECG diagnosis, a positive CDG can indicate underlying cardiac abnormalities that the ECG cannot identify (PPV: 81.91%) and help direct patients to undergo CCTA early.

In this study, the SHI representing the spatial dispersion and the THI representing the temporal dispersion were calculated separately, and CDG value was generated using machine learning algorithm of linear kernel SVM. Each cardiac beat in the CDG graph represented the change rule of each ST‐T segment signal in the ECG vector diagram, thus the dispersion of the CDG represented the consistency of the changes in each ST‐T segment signal of the ECG vector diagram. CDG value was correlated with the degree of temporal dispersion, the larger the CDG value, the more scattered the CDG graph and the less consistent changes in the ST‐T segment of each cardiac cycle. Conversely, the smaller the CDG value value, the more regular the CDG graph and the stronger the consistency of the ST‐T segment of each cardiac cycle.^22^ The significant changes in ST‐T segment of ECG do not necessarily mean that the inconsistency of ST‐T segment changes in each cardiac cycle are enhanced, and many other factors can also lead to significant changes in the ECG signals. Therefore, significant ECG changes may not necessarily lead to CDG positive. In this study, only few (64/139, 46.04%) of the patients with significant ECG changes had a final diagnosis of CAD. The diagnostic performance of CDG in patients with suspected CAD with ECG significant changes needs further verification and improvement.

CAD has a high prevalence worldwide, and to date, CCTA has been considered a preferable, reliable noninvasive examination method for diagnosing this disease.^27^ However, massive numbers of patients with suspected CAD who have undergone CCTA later showed negative results.^3^ In our study, the CDG had a favorable sensitivity and specificity in diagnosing CAD, demonstrating that it can identify CAD patients and be used as a screening tool before the CCTA examination, thereby reducing unnecessary radiation exposure. Regarding early studies of the CDG, Dong et al.^32^ included 86 patients, using single‐photon emission computed tomography myocardial perfusion imaging as the reference standard, and showed that the CDG had an accuracy of 84.9%, sensitivity of 84.0%, and specificity of 89.4% in the diagnosis of myocardial perfusion abnormalities. Wen et al.^33^ recruited 17 middle‐distance runners aged 14−28 years to assess the feasibility of the CDG for detecting cardiac function in exercisers and showed that the accuracy of the CDG was 80.0% when the levels of creatine kinase MB (CK‐MB) isoenzyme and high‐sensitivity troponin I (hsTnI) were used as the reference standards. Sun et al.^31^ measured the CDG in 499 patients with negative findings on 12‐lead ECG and achieved a diagnostic accuracy for myocardial ischemia of 91.1% when ICA stenosis ≥50% or Thrombolysis in Myocardial Infarction flow grade <3 were used as the positive reference standards. We also analyzed a subgroup of patients (*n* = 223) with negative diagnoses by 12‐lead ECG. The results similarly showed that the CDG had a high accuracy (80.27%) in diagnosing CAD. In addition, we are the first group to evaluate the CDG using hemodynamic parameters as a reference standard with impressive diagnostic performance (accuracy: 78.73%; sensitivity: 77.12%; specificity: 79.90%), which is higher than that when CCTA alone is used as a reference standard (accuracy: 71.55%; sensitivity: 72.80%; specificity: 70.89%). This outcome can be explained by the fact that functional abnormalities do not necessarily occur when there is an obstructive lesion in the coronary arteries.^6^, ^13^, ^19^ Hence, the CDG reflects functional information about the heart by detecting its electrical activity.

Because the ICA examination is the gold standard for diagnosing CAD,^34^ we analyzed 13 patients who underwent an ICA examination after CCTA and ECG. The results showed that the diagnostic performance of the CDG was satisfactory, this conclusion is consistent with the findings of previous studies.^22^, ^32^, ^33^, ^35^ We further confirmed that the CDG had better diagnostic performance in diagnosing CAD and can be used as a screening tool before the CCTA examination in patients with suspected CAD. We discovered that positive CAD patients had higher CDG values than negative CAD patients, and there was a correlation between CDG values and CT‐FFR, although the correlation was weak (*r* = −.395, *p* < .001). In patients with suspected CAD, especially those with a negative ECG diagnosis, higher CDG values indicate a greater likelihood of obstructive stenosis or hemodynamic abnormalities in the coronary arteries, thus suggesting that these patients should be further examined as early as possible.

Several limitations of this study should be noted. First, this is a single‐center retrospective study, and the diagnostic performance of the CDG needs to be confirmed further in a multicenter study with a larger sample size. Second, most of the included population did not meet the criteria for ICA testing, and so ICA or invasive FFR were lacking as a gold standard for validating the diagnostic performance of the CDG, even though many studies have shown that the CT‐FFR has equal diagnostic performance to the invasive FFR.

## 5 CONCLUSIONS

The ECG‐based CDG generated by deterministic learning can efficiently distinguish CAD and non‐CAD. Additionally, this performance was verified with different reference standards and subgroup analyses. This method can be used as a rapid and reliable noninvasive screening tool for suspected CAD patients before the CCTA examination, prompting potential positive patients to undergo further testing early or to reduce unnecessary CCTA testing.

## AUTHOR CONTRIBUTIONS

Ying Wang carried out the study, participated in the study design, and wrote the final manuscript. Jiaxin Sun carried out the study and participated in its design and in drafting of the manuscript. Weihua Zhao contributed to the managed the literature search. Lin Li participated in the interpretation of data. Cong Wang, Xinxin Yu, Kui Sun, and Hui Gu participated in the study, and interpretation of data. Qinghua Sun and Ximing Wang conceived the study. All authors read and approved the manuscript.

## CONFLICT OF INTEREST STATEMENT

The authors declare no conflict of interest.

## Supporting information

Supporting information.

## Data Availability

The data sets of this study are not publicly available due to privacy hospital policy but are available from the corresponding author on reasonable request.
