# Supplementary material for: ECG‐based cardiodynamicsgram can reflect anomalous functional information in coronary artery disease
Source: Clin Cardiol. 2023 Apr 6;46(6):639–47. doi: 10.1002/clc.24019 (PMC10270272; doi:10.1002/clc.24019)
Supplement: Supplementary file 1 — Supporting information. [file CLC-46-639-s001.docx]

**Supplementary Table 1. Diagnostic performance of CDG in patients with a negative standard 12-lead electrocardiogram diagnosis**

|  | CCTA<50% | |  | CCTA≥50% | | Total |
| --- | --- | --- | --- | --- | --- | --- |
|  | CT-FFR>0.8 | CT-FFR≤0.8 |  | CT-FFR>0.8 | CT-FFR≤0.8 |  |
| CDG <0 (%) | 102 (45.74) | 2 (0.90) |  | 3 (1.34) | 22 (9.87) | 129 (57.84) |
| CDG ≥0 (%) | 17 (7.62) | 20 (8.97) |  | 6 (2.69) | 51 (22.87) | 94 (42.15) |
| Total (%) | 119 (53.36) | 22 (9.87) |  | 9 (4.04) | 73 (32.74) | 223 |

Data are presented as frequencies and percentages as appropriate.

Abbreviations: CCTA, coronary computed tomography angiograph; CT-FFR, computed tomography-derived fractional flow reserve; CDG, cardiodynamicsgram.

**Supplementary Table 2. Repeatability test of data measurement**

|  | ICC | 95%CI | P |
| --- | --- | --- | --- |
| CT-FFR_LAD_ | 0.979 | 0.972～0.985 | <0.001 |
| CT-FFR_LCX_ | 0.981 | 0.959~0.990 | <0.001 |
| CT-FFR_RCA_ | 0.978 | 0.955~0.988 | <0.001 |

Abbreviations: CDG, cardiodynamicsgram; CT-FFR, computed tomography-derived fractional flow reserve; ICC, interclass correlation coefficient; CI, confidence interval.


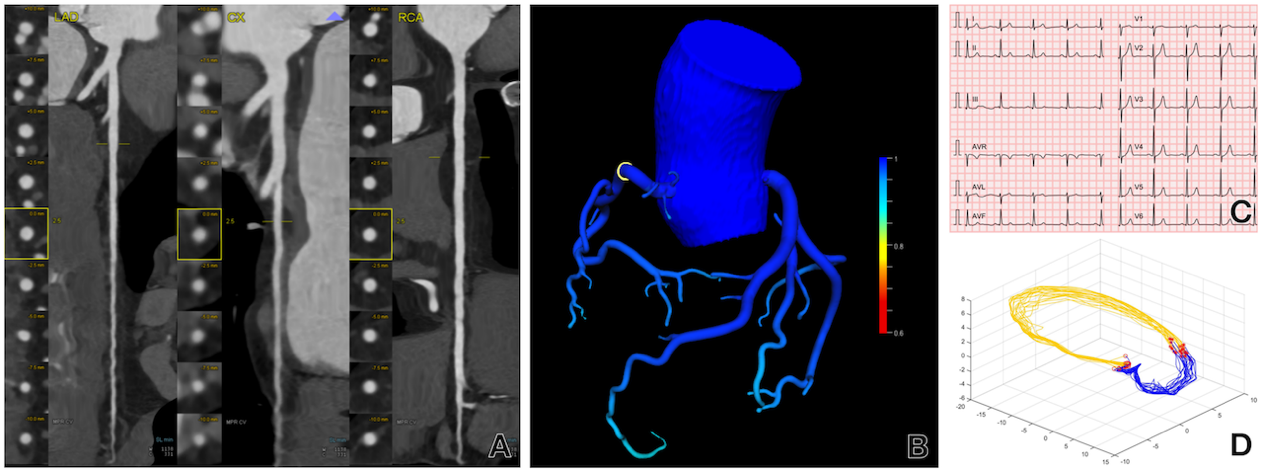


**Supplementary Figure 1. Data from a 64-year-old male patient with no stenosis on coronary computed tomography angiograph (CCTA) and no computed tomography-derived fractional flow reserve (CT-FFR) abnormalities.**

**(A)** Curved plannar reconstruction of the left anterior descending artery, left circumflex artery and right coronary artery. **(B)** The image shows CT-FFR >0.8 for all branches ≥1.8 mm. **(C)** The standard 12-lead electrocardiogram shows no abnormalities. **(D)** The cardiodynamicsgram (CDG) shows a regular shape in a 3D graph, and the CDG value is -7.20.


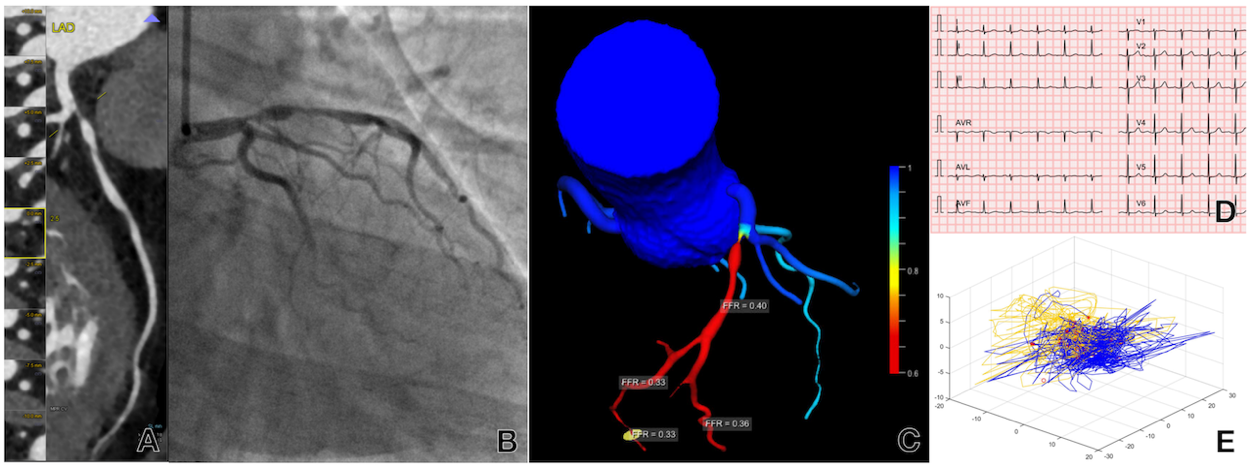


**Supplementary Figure 2. Data from a 39-year-old female patient with left anterior descending artery (LAD) showing an obstructive lesion on coronary computed tomography angiograph (CCTA) and an abnormality on computed tomography-derived fractional flow reserve (CT-FFR).**

**(A)** Curved plannar reconstruction of the LAD (severe proximal stenosis). **(B)** Corresponding projection of the invasive coronary angiography confirming high-grade stenosis. **(C)** The image shows a CT-FFR <0.8 at the distal end of the LAD. **(D)** The standard 12-lead electrocardiogram shows no abnormalities. **(E)** The cardiodynamicsgram (CDG) shows an irregular shape in a 3D graph, and the CDG value is 4.39.
